# Local knowledge held by farmers in Eastern Tyrol (Austria) about the use of plants to maintain and improve animal health and welfare

**DOI:** 10.1186/s13002-016-0104-0

**Published:** 2016-09-12

**Authors:** Christian R. Vogl, Brigitte Vogl-Lukasser, Michael Walkenhorst

**Affiliations:** 1 Working Group: Knowledge Systems and Innovation, Division of Organic Farming, Department for Sustainable Agricultural Systems, University of Natural Resources and Life Sciences (BOKU), Vienna, Austria; 2 Departement of Livestock Sciences, Research Institute of Organic Agriculture (FiBL), Ackerstrasse 113, Frick, 5070 Switzerland

**Keywords:** Ethnoveterinary medicine, Traditional ecological knowledge, Local knowledge, Organic farming, Animal feed, Animal husbandry, Preventive veterinary medicine

## Abstract

**Background:**

The sustainable management of animal health and welfare is of increasing importance to consumers and a key topic in the organic farming movement. Few systematic studies have been undertaken investigating farmers’ local knowledge related to this issue. Ethnoveterinary medicine (EVM) is a discipline focusing on local knowledge and folk methods in veterinary medicine, however most ethnoveterinarian studies primarily address the treatment of animal diseases. Very few studies have explored prophylactic methods.

**Methods:**

An ethnoveterinary research project in Eastern Tyrol (Austria) was conducted in 2004 and 2005 to gather information about local knowledge of animal husbandry from 144 informants, with the emphasis on plants that maintain livestock health and welfare.

**Results:**

Informants mentioned a total of 87 plants and 22 plant-based generic terms in the context of maintaining and improving livestock health and welfare. The most important preventive measures for maintaining and improving animal health and welfare were practices related to “fodder” and “feeding”. In this category the plants mentioned could be grouped according to three different perceptions about their effect on animals: “Good or bad fodder”, “Functional fodder” and “Fodder medicine”. In addition to fodder, environmental management, the human-animal relationship, household remedies and cultural/religious activities were also mentioned. When asked about practices in the past that maintained animal health and well-being, interviewees mentioned, for example, the importance of the diversity of sources that used to be available to obtain feed and fodder.

**Conclusions:**

The informants’ approach that feeding is central to livestock welfare is in line with the standard scientific literature on animal health, including in organic farming. Various scientific studies into common fodder evaluate the nutritive and dietary value, efficiency and safety of fodder. Future studies also have to consider the evaluation of traditional, local fodder resources. In fact, the value of ‘food as medicine’ for humans in the context of local knowledge has been widely assessed, but the potential health benefits of fodder and nutraceuticals in local and traditional ethnoveterinary methods require further attention.

## Background

In industrialised countries, the recent expansion of organic farming and restrictions in the use of allopathic medicine, as well as frequent discussions in the media and society at large about animal welfare, have shown the growing interest among stakeholders in sustainable management of animal health and welfare. In particular the Council Regulation concerning organic production [1] and its amendments clearly describe methods for assuring animal health on organic farms. According to this regulation, the priority is on keeping livestock healthy through breeding and management measures (including feeding and housing). In the event of disease, “phytotherapeutic, homeopathic and other products” shall primarily be used as therapeutic measures, with “chemically synthesised allopathic veterinary medicinal products” as a last resort and limited in the frequency of their application. Moreover the thematic priority of the current animal health legal framework of the European Union and the World Organisation for Animal Health [2] is “prevention is better than cure”. This approach also meets consumers’ demand for high-quality animal food products and responds to increased public interest in the way in which livestock are treated [3–5].

There is a wide variety of approaches available to implement concepts of sustainable animal health and welfare management, including diagnostic tools of preventive veterinary medicine, advice to farmers about health and management [6], structured exchanges of farmers’ experiences in what are known as “farmer field schools” [7, 8] for example, and complementary medicine [9]. In addition to these approaches, gathering information about the existing knowledge held by farmers and their practices around health and welfare management would improve the understanding of farmers’ views and practices on this topic.

Local knowledge and folk methods based on plants are usually studied by ethnobotanists [10, 11] or scholars of ethnoveterinary medicine [12]. Ethnoveterinary medicine (EVM) is a discipline that focuses on local knowledge or folk methods concerning the prevention and cure of animal diseases [13, 14].

Today in many rural, developing countries where animal production plays an important role, EVM remains essential to people’s livelihoods for financial (lower costs) and practical (higher accessibility) reasons [15, 16].

In European countries, modern veterinary practices are common and there is a risk of EVM disappearing altogether [17, 18].

Very limited specific scientific research on EVM at a European level has been undertaken, with a few exceptions [17, 19–30], although it is becoming increasingly important in organic farming (see also the comprehensive review on European ethnoveterinary research by Mayer et al. [31]).

The term EVM is often equated with the concept of therapy or herbal remedies and may suggest only the use of medicines. Indeed, most ethnoveterinarian studies primarily address the treatment of animal diseases with local remedies, especially botanicals, while far fewer studies have featured prophylactic methods [13, 14, 16].

This paper is based on data from a research project designed to show local knowledge of therapy and medicine. However, the quantity and diversity of the information about ways of maintaining and improving animal health and welfare through preventive actions were unexpected. Due to the practice of extensive agriculture and the historical form of land use, local knowledge still exists in the study area about plant-based fodder, which contributes to animal health. The authors of the present study believe there is merit in these results being presented, even though the data was collected in 2005. The information about local knowledge that was collected remains relevant because farmers’ experiences over generations are still valuable, and will possibly be of even greater value in future. The US regulation on organic farming (National Organic Program, NOP), for example, includes a full ban on antibiotics in organic animal husbandry, although the EC Regulation for Organic Farming is less strict. The debates within the Transatlantic Trade and Investment Partnership (TTIP) have led to a move within the organic farming movement to tighten European regulations as well, which would result in an urgent need to search for alternatives. In support for this search for alternatives and to implement concepts of sustainable animal health and welfare, especially for the organic farming movement, there is an urgent need to gather information about current practices and local knowledge of animal husbandry, emphasising prophylactic methods with regard to animal health and welfare. Therefore this paper was based on the following research questions:

- What knowledge do farmers have of general practices that maintain the health and welfare of livestock?
- What knowledge do farmers have about plants that maintain the health and welfare of livestock?

## Methods

### Sampling

In the years 2004 and 2005, 144 informants from 16 communities in Eastern Tyrol were interviewed by means of three free lists based on purposive sampling and snowball sampling [32]. Informants were aged between 33 and 93 (mean: 62 years of age). Seventy-five were female, 69 were male. The farms surveyed were situated between 670 and 1,600 m a.s.l. Each of the studied farms keeps differing numbers and types of animals: some may have all of these animals or just specialise in one type e.g. cattle only. On average, the studied households keep 18 cattle (119 farms), 47 sheep (23 farms), 6 goats (20 farms), 4 pigs (89 farms), 28 hens (69 farms) and 2 horses (16 farms). Farming is combined with different kinds of off-farm labour, with 69 farms managed part-time and 47 full-time (28 informants did not want to provide information about this). According to the farmers, federal subsidies under the Austrian Environmental Programme for Agriculture make up an important part of their income.

### Data collection

Data collection was based on three free lists [32]. These were:

- Free List 1 (FL 1, *n* = 144): The informants’ knowledge of general practices that maintain the health and welfare of livestock
- Free List 2 (FL 2, *n* = 144): The informants’ knowledge of specific plants that maintain the health and welfare of livestock
- Free List 3 (FL 3, *n* = 144): The informants’ knowledge of plants that treat livestock diseases. The informants also mentioned plants related to maintaining livestock health and welfare here. Only this data is presented in this paper.

For all the free lists, semi-structured interviews were also conducted about the respondents’ knowledge of the use of the plants mentioned (*n* = 144).

In 2005, a semi-structured interview was conducted with five of the most knowledgeable respondents from that sample, referred to here as key informants, about the history of fodder and feeding, as the analysis showed these to be key aspects in maintaining livestock health and welfare (*n* = 5 from the above mentioned sample of 144 respondents). Four of the five key informants were male and one was female. The five informants were aged between 48 and 78 and all were farmers. One informant also worked as a forest ranger. The period of time covered in these interviews was from 1940 to 2005 approximately.

For the purposes of this paper, the term ‘plant’ is used for plants that are classified as a single taxon, plants identified at genus level and lichens. In addition, plants that were purchased as processed commercial product are identified by their product name (apple cider vinegar, red and white wine, peppermint oil, coffee, homeopathic medicine, black tea). Plant-based material based on various plants is summarised in generic terms (e.g. mountain meadow hay).

To identify the plant taxa, checks were first undertaken to establish whether the plants actually grow in the study area. The scientific name for the plant name mentioned was then identified based on standard botanical literature and the finding cross-checked with the informants based on pictures [33–35]. The authors, some of whom have botanical research experience in the study area, are confident that the recorded plants are plant taxa that are well known in the region. In cases in which plants could not be related by the informants to one single plant taxon, the plants are reported here at genus level only.

Before the interview commenced, a detailed written explanation of the project, including an abstract and contact information for the authors and their affiliation, was given to the respondents. Verbal consent for further inquiry was obtained. The interview was recorded if the informants agreed to it; if not, their answers were noted down. Whenever possible and permitted, photographs were taken. Photographs and audio recordings are deposited at the University of Natural Resources and Life Sciences in Vienna (BOKU). The collected data was stored, categorised and analysed in an MS ACCESS (Microsoft Inc. 2007) database.

### Characteristics of the study area

**Fig. 1:**
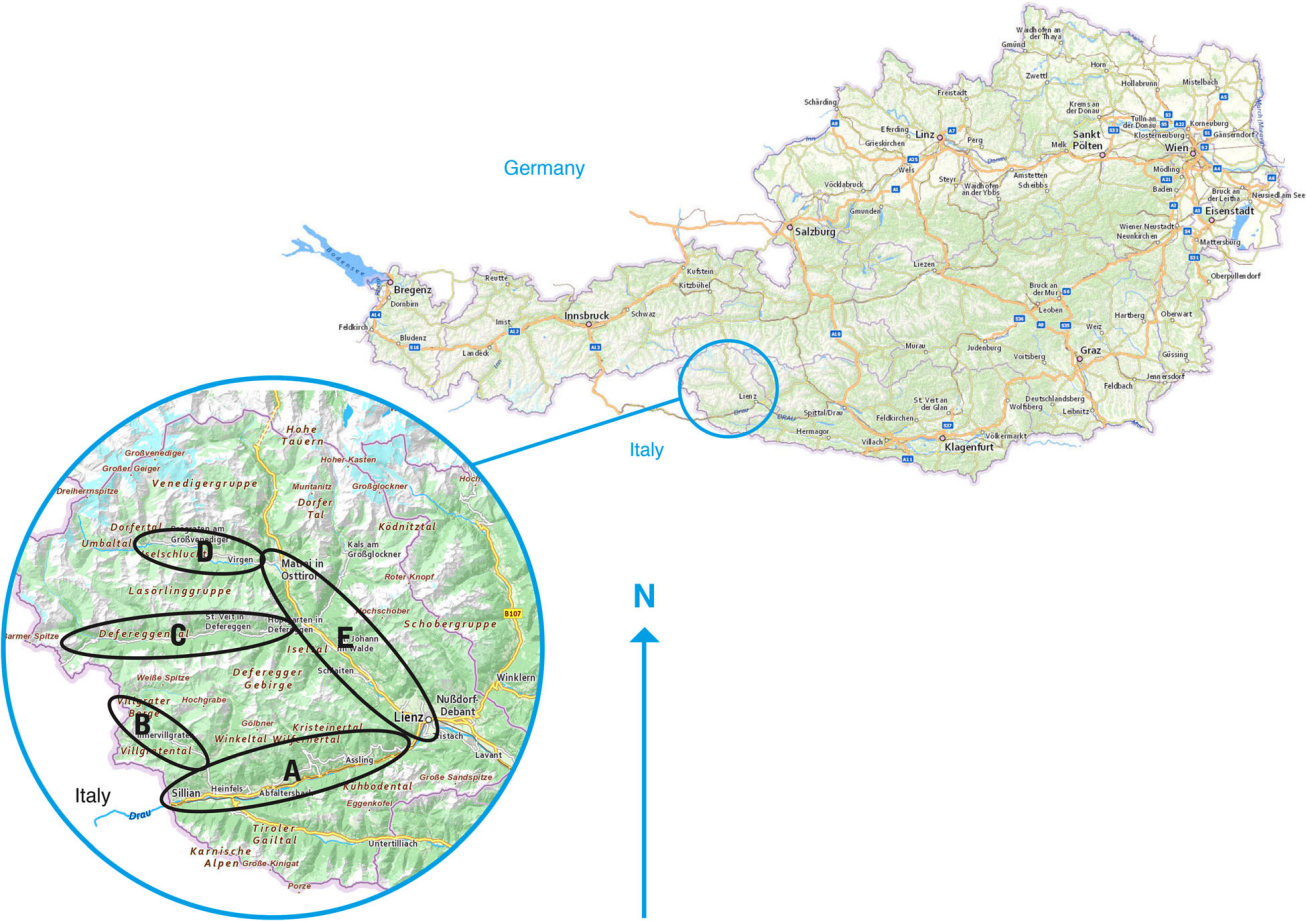
Map of Austria (upper right) and map of Eastern Tyrol (lower left). Circles indicate the valleys where the interviews were done (A: Drautal, B: Villgratental, C: Defferegental, D: Virgental, E: Iseltal). Scale: distance Sillian – Lienz approx. 27 km: (Source: basemap.at 2016)

The district of Lienz (Eastern Tyrol) is located in the Austrian part of the Eastern Alps (Fig. 1), the highest peak of which is the Grossglockner (3,797 m). The region has an area of 2,020 km^2^ and is home to 49,000 inhabitants living in 33 villages [36]. In Eastern Tyrol, 1,675 farms are managed by families; an additional 235 farms are managed by associations of varying legal status [37]. The study area includes the mountain range of the Hohe Tauern, which contains a national park. The large altitudinal gradient from 600 m to almost 4,000 m above sea level gives rise to a narrow sequence of different natural and agricultural zones. At the lowest level, the natural vegetation is deciduous and mixed forests characterised by beech (*Fagus sylvatica* L.) and fir (*Abies alba* Mill.). However, these forests have only survived in small enclaves due to the huge changes made by humans over a long period of time. Spruce (*Picea abies* (L.) H. Karst.) forests start at 1,000 m a.s.l. and extend to about 1,700 m a.s.l., before being replaced by open woods with larch (*Larix decidua* Mill.) and mountain pine (*Pinus mugo* Turra) at elevations of about 2,100 m a.s.l.. Alpine pastures are located up to 2,500 m a.s.l. Above the treeline, dwarf shrubs form a transition to the high alpine grass formations and lichens at the upper limit of vegetation [36]. Annual precipitation in the region is 826-1,354 mm and the mean annual temperature is 2.8-6.9 °C (values depend on exposure and altitude). This broad range of natural conditions within a small area has led to a highly diverse pattern of human- environment relationships [38]. Adaptive management of natural resources by Alpine small farmers has created a typically diverse and multifunctional landscape. The historical form of agriculture in this region can be described as “mountain cereal grazing [39]” where the farming of arable land (up to 1,700 m a.s.l.) for cereal cultivation, field vegetables, fibre crops etc. and the farming of a high diversity of animal species, with a low number of individuals per species, were the main components of the subsistence system until the 1970s [40].

### History of feed and fodder in the study area

According to the key informants, most of the cultivated land in close proximity to the homesteads was used for the production of food in arable farming. A patchwork of plots from lowland to highland was required to feed the animals not just with fresh, green fodder during the vegetation period, but also to provide fodder for storage and feeding during the winter. A great variety of pastures with different systems of grazing existed. Communally and individually-owned resources distributed over different vegetation levels allowed the farmers to maximise the period of grazing between spring and autumn. The animals were kept on open areas, such as steep slopes, wood pastures and alpine grazing grounds. Livestock movement by shepherding (especially for sheep) was widespread, and the use of plots and the timing of this use were regulated through informal and formal institutions.

During the summer (June - September), almost all the animals were transferred to the high Alpine grazing grounds, accompanied by some members of the farmer’s family. During this time, only a few animals were kept at the homestead, such as a cow or a goat, to provide milk for family members who had to attend to the remaining business at the homestead.

Acquisition of fresh (as distinct from grazing) and dried fodder was distributed over a diverse range of plots and carried out with different management practices. Mowing of grassland near the homestead was designated to habitats where tillage was not possible. Therefore, only boundary areas, areas with poor soils, wet and sedgy grassland and steep slopes were designated to produce grass hay. Most winter fodder was produced on alpine meadows at elevations of at least 1,900 m above sea level. The informants stressed that given the steepness of most of these slopes, this was always hard labour performed by hand. Mowing and removing the cuttings was not only labour intensive, but extremely dangerous as well. For example, the hay had to be carried on people’s backs to a barn situated near the meadow, and then transported to the homestead in winter on special sledges.

Another traditional and important fodder resource included leafy fodder and leaf hay from trees and shrubs. Single deciduous trees were therefore grown near the farmhouse or hedgerows which, combined with deciduous trees, served as boundaries to neighbouring plots. On erodible slopes, trees and meadows were combined not only to produce leaf hay, but also for soil conservation purposes. Trees such ash (*Fraxinus excelsior* L.) were pollarded. Shrubs such as hazel (*Corylus avellana* L.) were felled at ground level with the intention of promoting basal shoots for harvest in future. Branches were bunched and dried or sometimes the foliage was fed fresh. Pollarding and thinning out hedgerows was not only necessary to produce leaf hay, but also to limit the number and size of trees and shrubs so as not to inhibit the growth of crops or grass in enclosed fields. The twigs of plants such as raspberries (*Rubus idaeus* L.) were also collected in the summer on the edges and glades of woods. They were cut as a whole, tied together in bundles, and dried.

A common practice in conifer stands was not only to gather needles from the forest floor for use as bedding for livestock (mainly from larch *L. decidua*; local term: *Streibekotto*), but also to pollard conifers such as common spruce (*P. abies*). A special meal was produced (stamped or ground) from the dried spruce needles and used as an addition to cattle fodder, known locally as “black concentrate feed” (local term: *schwarzes Leck*).

Particular wild fodder plants were frequently gathered in various alpine habitats (e.g. different taxa of dock and thistles, common juniper, Iceland moss). In ruderal habitats, plants such as stinging nettles or docks were collected frequently. This fodder was either fed directly to livestock or gathered for winter fodder.

Crop residues and by-products from all cultivated plants were common traditional fodder resources. High-quality crop products fit for human consumption were rarely used. Such feedstuffs were not entirely excluded, but the proportion was very reduced and only utilised in particular periods (e.g. *Linum usitatissimum* L. prior to breeding animals).

In the past in the study area, it had been essential for all of the ‘weeded’ plants from cultivated crops or from home gardens to be used as fodder (local term: *Gross*) for goats, pigs and even for cows. The term ‘weed’ was not used at all in the past, but rather the term *Gross* (grass), emphasising its former use as fodder. This weed fodder was the only payment women received for weeding the fields of large farmers. However, not only the weeded plants as a whole, but also specific taxa were mentioned as being essential for subsistence. Common chickweed (*Stellaria media* (L.) Vill.), for instance, used to be popular for feeding to chickens and pigs ([40, 41] for further details on the use of “weeds”).

Not only were the places from which fodder was acquired multifaceted and the composition of the fodder itself diverse, but there were also many methods for processing feedstuff. Processing ranged from reducing particle size by simply cutting the pieces, grinding, chaff cutting or squashing all the way to brewing, soaking and boiling. Informants pointed out that this made the fodder more digestible and tasty, and even improved the nutritional value of the crop residues, for example, which are said not to be particularly nutritious or palatable. Cut or ground feedstuff allowed an easy mixing of different fodder resources too. Processing was undertaken on a daily basis, and the composition of daily feed rations was adapted not only to different animal species but also to their different roles (e.g. working animals, pregnant animals etc.). The use of externally produced fodder imported to the region was not common. Although in the past it used to be hard to feed the animals due to fodder shortages, the different kinds of fodder used back then were said to be very healthy. The informants’ perception is that in the past few decades there has been a fundamental shift in animal nutrition.

The basic changes in the land use system are abandonment of arable farming and specialisation in grassland with higher livestock productivity and increased mechanisation. At the same time, arable land near the homestead is being converted into permanent meadows where hay is produced for winter fodder. The grass plots are fertilised regularly with liquid (slurry) or solid manure. An increasing practice is the renovation of grassland with improved, commercially available grass/legume cultivars to raise the green-matter yield. With these activities, generally high yields with poor diversity can be achieved. The higher alpine zones are dominated by pastureland, where animals (mainly cattle and sheep) remain throughout the summer.

The scarcity of manpower caused by an exodus of the rural population and the enormous rise in labour costs due to fundamental economical and political changes result not just in farm mechanisation wherever possible, but also to a decline in labour-intensive traditional techniques such as tree pruning or making alpine hay on steep slopes where mechanisation is not possible. Most former hay-making areas on higher elevations have been converted into extensive pastures or secondary fallow, and the typical treetops of pollarded trees are no longer part of the cultural landscape. Traditional shepherding techniques have for the most part been replaced by large-scale free-range grazing. The practices of gathering fodder plants, feeding weed-fodder to livestock or pollarding conifers have almost all declined.

Nevertheless, when compared with intensive livestock production, which is characteristic of various European areas where cows are mainly fed silage, second and third-growth crop and concentrated feedingstuffs, livestock production on farms in Eastern Tyrol can still be described as extensive agriculture, which is typical for marginal areas in Europe.

## Results

### Knowledge of general practices

The most important preventive measures in maintaining and improving livestock health and welfare are practices related to fodder and feeding, followed by practices related to management measures. Management measures can be divided into management directly related to the animal (animal care or management) and management of the animals’ environment (environmental management), especially in animal housing (Table 1).

**Table 1:** Categories and number of practices mentioned per category in relation to the farmers’ aim of maintaining and improving livestock health and welfare (*n* = 144; 1,139 practices mentioned)

Category	Number of practices mentioned	Examples of practices mentioned by informants
Fodder/feeding	416	Kind of fodder, quality of fodder etc. (Table 2)
Animal care or management	338	Claw trimming, animal cleanliness, exercise during summer on alpine grazing grounds
Environmental management	209	Ventilation in the stable, cleanliness of troughs, sufficient litter
Human-animal relationship	70	Taking one’s time, handling with care and love, proper observation of animals
Veterinary medicine	21	Deworming, sheep dip, testing for Lyme disease, veterinarian medical control
Household remedy	18	Lubrication with used grease or lard against insects
Breeding	18	Own breeds, native breeds, Simmental breed vulnerable to claw problems
Cultural-religious activities	13	Sprinkling with holy water, feeding of sacred Easter horseradish, feeding of sacred salt
Other categories	36	Organic farming

Within the category fodder/feeding, the quality of the fodder (mentioned 125 times) and the kind of fodder given to the animals (mentioned 116 times) were the most frequent sub-categories mentioned (Table 2). Concerning “quality of fodder”, informants mentioned for example that fodder given to the animals on a daily basis, such as roughage, has to be well dried, clean and not mouldy, discoloured or soiled. Good harvesting and storage of fodder are essential. The best meadow grasses come from nitrogen-poor swards with a large quantity of “herbs”. This fodder is said to be appetising, easily digestible and therefore healthy for the animals. Informants also pointed out that clean water is essential for the animals’ good health and that this water should have the quality of potable water.

**Table 2:** Sub-categories from the “feeding/fodder” category (Table 1), categorised and sorted by the authors by coincidences in contents (*n* = 144; 16 informants with no answer in this category; 1 questionnaire not analysable; 416 practices mentioned in total)

Sub-Category	Number of practices	Explication	Examples of practices
Kind of fodder/feed	164	Composition of fodder and different components of particular fodder, including feeding composition at a particular stage of animal life	“mountain meadow hay”, “wheat bran”, “colostrum” …
			“14 days before calving, feeding of …”
Quality of fodder	125	Different terms for high quality	“clean”, “healthy”, “good”, “own”
Feeding ration	65	Amount of feeding ration and appropriate rate of different components of the fodder	“more hay than silage“, “enough of…”, “not too much of …”
Method of fodder production	39	Methods concerning mainly grassland	“no artificial fertiliser for grassland”, “mowing after sunset”, “mowing when grasses are mature”
Other sub-categories	23		“naturally feeding”, “organic agriculture”

Concerning the “kind of fodder”, the informants mentioned plant-based fodder but also other feedstuffs such as colostrum, mineral supplements and salt. Different “feeding compositions at particular stages” of the animals’ development are mainly offered prior to birth and after birth (see the chapter on fodder medicine).

With regard to “feeding rations”, informants spoke about the negative impact of the massive uses of silage or feeding concentrates on animal rations and welfare, and reported digestive disorders and reduced fertility. In the sub-category “method of fodder production”, informants stressed different techniques for obtaining high quality fodder. These included mowing after sunset or mowing when grasses are mature.

### Knowledge of plants that maintain livestock health and welfare

A total of 87 plants and 22 plant-based generic terms were documented related to maintaining livestock health and welfare (FL2, FL 3, key informant interviews) (Table 3). Of these, 51 different plants and a total of 16 plant-based generic terms were mentioned in FL 2.

**Table 3:** Categories and number of plants/generic terms per category in relation to the farmers’ aim of maintaining animal health and welfare in the free lists FL2, FL3 and in interviews on history of fodder and feeding (HI, *n* = 144, including 5 key informants). Most of the plants and generic terms show various uses and are mentioned in more than one category

Category	Number of plants mentioned				Number of generic terms mentioned			
	All	FL2	FL3	HI	All	FL2	FL3	HI
Total^a^	87	51	39	69	22	16	7	14
Kind of fodder/feed^a^	77	45	38	60	20	15	6	14
Care of the animals^a^	2	2	1	1	0	0/0	0	0
Environmental management^a^	11	10	5	10	4	3	2	3
Human-animal relationship^a^	0	0	0	0	1	0/0	0	1
Veterinary medicine^a^	0	0	0	0	0	0	0	0
Household remedy^a^ (excluding fodder medicine)	9	9	8	8	3	2	1	3
Cultural/religious activities^a^	17	8	10	17	5	4	1	4

^a^Multiple answers occurring between FL 2, FL 3 and HI

For the treatment of livestock diseases, a total of 98 different plants were known to be useful (FL 3; plants not shown here). Of these 98 plants, 39 are not only used to treat diseases, but are also used preventively to maintain livestock health and welfare.

With regard to the history of animal husbandry, 69 plants and 22 generic terms were mentioned.

The most frequently cited plants were linseed (*L. usitatissimum*), Iceland moss (*Cetraria islandica* (L.) Ach.), common spruce (*P. abies*) and wormwood (*Artemisia absinthium* L.). The most frequent generic terms were mountain meadow hay (*Bergheu*), hay-blossoms (*Heublumen*), herbs (*Kräuter*) and hard liquor (*Schnaps*). These plants and generic terms were all part of the “fodder/feeding” category. They are traditional fodder resources, with the key aspect that they have a diverse range of uses for animal health and welfare.

#### Kind of fodder/feed

Informants listed 77 plants and 20 generic terms that can be attributed to the “kind of fodder/feed” category, to which 55 plants and 14 generic terms were exclusively attributed. Informants’ perceptions of the degree of the relationship between fodder and livestock health and welfare were diverse.

The plants mentioned could be grouped according to three different perceptions of the plants’ effects on animals. Almost all the plants were perceived in more than one group. This chapter only presents the structure for categorising fodder/feed. Details on their uses and examples are presented in the chapter on details of the knowledge of the kind of fodder/feed.

1. “Good or bad fodder”: 34 plants and 13 generic terms were perceived as being fodder of “good quality” and nine plants as fodder of “bad quality” in general (three plants were listed in the category “good” and “bad” quality, depending on their mode of administration; Table 4).

**Table 4:** Plants and generic terms mentioned as maintaining and improving the health of animals in Eastern Tyrol (*n* = 144)

Category	Local name	FL1 + FL2	FL3	HI	Fo/Fe	Animal	Application	Other
Plants								
Abies alba Mill.	Tanne	1	0	x	a/c/d	var	Ap; Dig; Goo; Is; Per; Rest;	Em
Achillea millefolium L.	Schafgarbe	3	12/22	x	b/c/d	go, var	Ba; Dig; Is; Rep2; Sed;	Ra
Alchemilla spp.	Frauenmantel	1	2/4	x	a/d	var, ca	Dig; Goo; Is; Rep1;	Ra
Allium cepa L.	Zwiebel	0	6/20	x	d	ca, sh	Rep2;	-
Allium sativum L.	Knoblauch	0	2/8	x	c/d	ca	Ap; Rep1;	-
Alnus alnobetula (Ehrh.) K.Koch	Lutterstaude	0	0	x	b	var	Ba;	-
Althaea officinalis L.	Eibisch	0	3/5	x	d	ca, var	Is; Rep2;	Ra
Anthriscus sylvestris (L.) Hoffm.	Wiesenkerbel, Rosskümmel	3	0	-	a/b	ca	Ba; Goo;	-
Armoracia rusticana P. Gaertn., B. Mey. & Scherb.	Kren	1	0	x	-	var	-	Ra
Arnica montana L.	Arnika	2	11/58	x	c/d	var, ca	Is; Rep2;	Hr, Ra
Artemisia absinthium L.	Wermut	0	24/39	x	c/d	ca	Dig; Is; Sed;	Ra
Avena sativa L.	Hafer, Hobo	15	6/15	x	c/d	ca, sh, ho	Goo; Per; Rep1; Rep2; Sed; Sk;	-
Beta vulgaris L.	Futterrübe, Runkel	8	0	x	a	ca, pi	Goo	-
Betula spp.	Birke	0	1/2	x	d	var	Is;	-
Brassica oleracea var. capitata L.	Kobis, Weißkraut	2	1/6	x	a/d	pi, ca	Goo; Is; Rep1; Rep2;	-
Brassica rapa var. rapa L.	Rübe, Herbstrübe	2	0/15	x	a/c/d	ca, pi	Goo; Is; Per; Ur;	-
Calendula officinalis L.	Ringelblume, Ringelrose	0	1/30	x	d	ca, var	Dig; Is	Ra
Cannabis sativa L.	Hanf	0	1/1	x	d	ca	Rep2;	-
Carlina acaulis L.	Silberdistel	0	0	x	a	ca, pi	Goo;	-
Carum carvi L.	Kümmel, Kümmelstaude	0	2/6	x	d	ca	Rep2;	-
Cetraria islandica (L.) Ach.	Goasstraube, Isländisch Moos	22	13/44	x	c/d	ca, sh, ho, pi	Is; Per; Rest; Rep1; Sk;	-
Cirsium spinosissimum (L.) Scop.	Einhacken	1	0	x	c	ca, pi	Per;	-
Coriandrum sativum L.	Koriander	1	0	-	d	ca	Dig;	-
Corylus avellana L.	Hasel	0	0	x	a	var	Goo;	-
Cucurbita spp.	Focknkürbis	0	0	x	a	pi	Goo;	-
Elymus repens (L.) Gould	Queckenwurzn	0	0	x	a	ca, sh	Fs;	-
Epilobium spp.	Weidenröschen	0	0	x	-	var	-	Ra
Equisetum arvense L.	Zinnkraut	1	0	-	a	var	Goo;	-
Fagopyrum esculentum Moench	Buchweizen	0	1/1	-	d	ho	Rep1;	-
Foeniculum vulgare Mill.	Fenchel	1	2/6	x	d	ca	Dig; Rep2;	-
Fraxinus excelsior L.	Esche	2	0	x	a/c	ca, sh	Goo;	Em
Geranium spp.	Storchenschnabel	0	4/4	x	d	ca	Rep1;	-
Helianthus annuus L.	Sonnenblume	0	0	x	c/d	var	Sed;	-
Heracleum sphondylium L.	Bärenklau, Bärentatze	0	0	x	a/b	ca	Ba; Goo;	-
Hordeum vulgare L.	Gerste	16	5/16	x	c/d	ca, sh, ho	Per; Rep1; Rep2;	Hr
Hypericum perforatum L.	Johanniskraut	1	2/12	x	d	var, ca	Is; Rep2;	Hr, Ra
Juglans regia L.	Walnuß	1	0/2	x	-	var	-	Em
Juniperus communis L.	Kranewitten, Wacholder	6	5/14	x	a/c/d	ca	Dig; Goo; Is; Per; Sed;	Em
Lamium spp.	Taubnessel	1	0	-	a	chi	Goo;	-
Larix decidua Mill.	Lärche	1	0/40	x	a	var	Goo;	Em
Ligusticum mutellina (L.) Crantz	Madaun, Goblitz, Mutterwurz	0	0	x	a	var	Goo;	-
Linum usitatissimum L.	Leinsamen, Hoorsomen, Linsat	34	22/49	x	c/d	ca, sh, ho	Dig; Per; Rep2; Sk; Sed;	Hr
Malva neglecta Wallr.	Kaspappel	0	0/9	x	c/d	ca	Dig;	-
Matricaria chamomilla L.	Kamille	1	27/100	x	c/d	var, ca, chi	Is; Rep1; Rep2; Sed;	Hr, Ra
Medicago sp.	Luzerne	1	0	-	a	var	Goo;	-
Melissa officinalis L.	Melisse	1	0	-	-	var	-	Em
Mentha spp.	Minze	2	0/1	x	d	var	Is; Sed;	Em, Ra
Papaver somniferum L.	Mohn, Mogn	0	1/4	x	d	ca	Rest;	-
Peucedanum ostruthium (L.) W.D.J. Koch	Meisterwurz	0	3/5	x	d	var	Is;	Ra
Picea abies (L.) H.Karst.	Fichte	19	4/26	x	a/c/d	ca, sh, go	Ap; Dig; Goo; Is; Per; Rest;	Em
Pimpinella anisum L.	Anis	1	1/3	-	d	ca	Dig;	-
Pimpinella saxifraga L.	Bockwurz, Bibernelle	0	0	x	c	chi	Is;	-
Pisum sativum L.	Erbse	1	0	-	a	ca	Goo;	-
Plantago lanceolata L.	Spitzwegerich	1	0	-	a	var	Goo;	-
Prunus avium L.	Kirsche	0	0	x	a	var	Goo;	-
Pteridium aquilinum (L.) Kuhn	Adlerfarn	0	1/1	x	d	sh	Ap;	Em
Quercus spp.	Eiche	0	0	x	a	var	Goo;	-
Ranunculus spp.	Hahnfuß	1	0	-	b	var	Ba;	-
Rhinanthus spp.	Klopf	1	0	-	b	var	Ba;	-
Rhododendron spp.	Almrose	0	0	x	b	var	Ba;	-
Rosa spp.	Rose	0	1/1	-	d	ca	Rep2;	-
Rubus idaeus L.	Himbeere	0	0/1	x	a	var	Goo;	Ra
Rumex spp.	Saupletschn, Focknpletschn, Sauer-Ampfer	8	0/1	x	a/b/c/d	ca, pi, ho, chi	Ba; Goo; Is; Per; Rep2;	-
Salix spp.	Palmbuschn	2	2/2	x	-	var	-	Hr, Ra
Salvia officinalis L.	Salbei	0	0/3	x	-	var	-	Ra
Sambucus nigra L.	Schwarzer Holler	3	2/16	x	c	ca, pi	Is;	Em, Hr
Secale cereale L.	Roggen	7	12/18	x	b/c/d	ca, pi	Ba; Rep1; Rep2;	-
Solanum tuberosum L.	Erdäpfel, Kartoffel	5	0/2	x	a/d	chi, ca, sh	Fs; Goo; Rep2;	-
Stellaria media (L.) Vill.	Hühnerdarm, Hiagepanze	0	0	x	a	pi, chi	Goo;	-
Symphytum officinale L.	Beinwell	1	0/2	-	a	chi	Goo;	-
Thymus spp.	Quendel	1	1/3	x	c/d	ca, Pi	Ap; Is; Per;	Hr
Trifolium spp.	Kleegras	2	0	-	a	ca	Goo;	-
Triticum aestivum L.	Weizen	14	2/8	x	c/d	ca	Dig; Rep2; Rest;	-
Urtica dioica L.	Brennessel	13	1/5	x	a/c/d	ca, pi, chi	Dig; Goo; Is; Per; Rest; Sk;	-
Usnea spp.	Baumbart, Rock	0	0	x	a	var	Fs;	-
Vaccinium myrtillus L.	Schwarzbeere, Heidelbeere	0	0/8	x	-	var	-	Ra
Verbascum spp.	Himmelsbrand	0	0	x	-	ar	-	Ra
Vicia faba L.	Scholleboan, Bühn	5	0	x	a	var	Fs;	-
Vicia sativa L.	Futterwicke	1	0	-	a	ca	Goo;	-
x Triticosecale Wittm.	Triticale	0	1/1	-	d	sh	Rep1;	-
Zea mays ssp. mays L.	Türgn, Mais	2	0/1	x	a	chi	Goo;	-
Processed commercial products								
Camellia sinensis (L.) Kuntze (black tea)	Schwarztee	0	4/41	x	d	ca	Is; Rest;	-
Coffea arabica L. (coffee)	Kaffee	1	17/29	x	d	ca	Rest; Rep2;	-
Echinacea spp. (homöopathic medicine)	Echinacea	1	0	-	-	ca	-	Hr
Malus domestica Borkh. (apple cider vinegar)	Apfelessig	4	5/10	x	c/d	ca, pi, var	Is; Rep1;	Ca, Em
Mentha spp. (peppermint oil)	Minzöl	1	0	-	-	ca	-	Ca
Vitis vinifera L. (red and white wine)	Wein	1	4/6	x	d	ca, go	Rep1; Rep2; Rest;	-
Generic terms								
“Alpine fodder”	Almgras, Almfutter	7	0	x	a	var	Goo;	-
After-grass	Gruimat	6	0	x	c	var	Rep2; Is;	Ra
Ash	Asche	0	1/1	x	d	pi	Rep1;	-
Beer	Bier	0	4/4	-	d	ca	Rest; Rep2;	-
Bread	Brot	0	0	x	d	ca, var	Rest	Hum, Ra
Concentrated feeding stuff	Leck, Kraftfutter	9	0	x	a/c	var	Goo;	-
Grass	Gras	4	1/4	-	a/c/d	ca, chi	Goo; Rep1;	-
Grist	Schrot	1	0	x	a	ca	Goo;	-
Hard liquor	Schnaps	0	39/84	x	d	ca	Rest; Rep2; Sed;	Hr
Hay	Heu	10	0/7	x	a	var	Dig; Goo; Is	Ra
Hay from marshy/mossy meadow	Sauerheu	3	0	-	a	ho	Goo;	-
Hay-blossoms	Bliuma, Mürach	35	3/10	x	a/c/d	pi, ca, chi, var	Goo; Is; Per; Rest; Rep2	Em, Hr, Ra
Herbs	Kräuter	14	0/1	x	a	ca, pi, var	Goo; Is;	Ra
Marc	Trester	1	0	-	a	ca	Goo;	-
Moss	Moos	0	1/1	x	-	pi	-	Em
Mountain meadow hay	Bergheu, Wiesenheu, Almheu	27	0/6	x	a/c/d	var	Dig; Goo;	-
Roughage	Raufutter	2	0	-	a	var	Goo;	-
Sawdust	Sägemehl	2	0	-	-	var	-	Em
Silage	Gärfutter	2	0	-	a	var	Goo;	-
Straw	Stroh	9	0	x	a/c	var	Fs; Rep2;	Em, Hr
Sugar	Zucker	0	3/6	x	d	ca	Rep2;	-
Thistles	Disteln	5	0	x	c	ca, pi	Per;	-

Legend

FL1 and FL2: Plants mentioned in Free List 1 and Free List 2. Figures are frequency of mention

FL3: Plants mentioned in Free List 3. First figure gives frequency of mention for preventive purposes. Second figure gives total frequency of all mentions of this plant being for preventive but also curative use. e.g. 22/49: Linum usitatissimum mentioned 49 times in total, 22 of these mentions were related to preventive use

HI: Plants mentioned in interviews (*n* = 144, including 5 key informants) on history of fodder and feeding (x = plants mentioned)

Fo/Fe: Plants mentioned in the category fodder/feeding: (a) good fodder; (b) bad fodder; (c) functional fodder; (d) fodder medicine

Animal (mentioned in the category fodder/feeding): chi = chicken; ca = cattle; go = goat; ho = horse; pi = pig; sh = sheep

Application: Lists only applications for preventive use for fodder/feeding, according to the information of the respondents as exemplified in the citations for possible applications below:

(Ap) Antiparasitics: preventive of intestinal parasites

(Ba) Bad quality in a general alimentary way: lowers quality of hay if too much represented in grassland, not to feed to swine to prevent swine erysipelas; weed

(Dig) Digestion: enhances and improves digestion, increases appetite, prevents diarrhoea, prevents disorders in digestion, avoids disorders in digestion by conversion of feeding especially for young stock, healthy digestive tract (especially after deworming) good for digestion while also calming, avoids metabolic disorders during conversion of feeding especially for young stock, stimulates digestion

(Fs) Fodder used in times of scarcity of fodder

(Goo) Good quality in a general alimentary way, variation in diet and eaten with pleasure (when fed fresh), rearing fodder for chicks, good fodder quality (palatable and good nutritive value only if harvested properly), appetising and easily digestible, good fodder quality if fed with caution and not looking too much at performance (negative when used in large quantities), freshly cut grass has good fodder quality if animals are unable to graze on pastures

(Is) Immune system: prevents bovine influenza, improves health and fitness in general, spring therapy for good health and to avoid iron deficiency, prevents swine erysipelas, good for strong immune system, generally blood cleansing, increases the body’s defences, in general tonic, rearing fodder for poultry to prevent diseases

(Per) Performance enhancer: promotes weight gain, growth and development, fattening fodder, increases production of milk, improves milk yield and milk fat, after birth to increase milk production, gives power, nutritious, improves laying performance, gives butter a nice colour

(Rep1) Reproductive 1: avoids milk fever, facilitates delivery, prevents retained placenta, nutritious for pregnant animals (in the final month before delivery), preparation fodder for birth, labour inducer and to induce uterine contractions, refreshment after delivery and to avoid circulatory disorders, helps with quick expulsion of the placenta, expands birth canal, avoids expulsion of uterus

(Rep 2) Reproductive 2: preparation fodder for improved oestrus and avoiding silent oestrus, good for female organs, given before the dams are inseminated (bred) for better acceptance of foetus, stimulates ovulation/oestrus inducer

(Rest) Restorer: restorer after diseases, restorer when animals look ill and have lost weight, restorer for weak animals, restorer after delivery, refreshment for calves after birth

(Sed) Sedative: to avoid irritated animals, to avoid stress after transport or being bought in

(Sk) Skin: improves skin, udder health and hoof quality, improves coat gloss, improves condition of eggshells

(Ur) For better urination

Other categories besides fodder/feeding: (Ca) care of the animals, (Em) environmental management, (Hr) household remedy, external application, in contrast to internal application as fodder medicine, (Hum) Human-animal relationship; (Ra) cultural/religious activities
2. “Functional fodder”: 25 plants and 7 generic terms were perceived as being fodder with a positive effect on health, natural resistance and/or performance. This kind of fodder could also be indicated as functional fodder ([42] used the term functional food). Functional food is characterised as having other effects on body functions besides their main nutritional or delight purposes. Fodder from plants mentioned in this survey as increasing the health, natural resistance and performance of livestock are given over a longer period of time and sometimes even on a daily basis (in contrast to fodder medicine).
3. “Fodder medicine”: 44 plants and 8 generic terms were perceived as being fodder to avoid disorders or diseases, and used as a preventive treatment (ingested in a “fodder context”) in order to obtain a specific medicinal action ([42] used the term food medicine). Fodder medicine is given anywhere from single administrations to up to a few days and on specific, discreet occasions only. Plants used as fodder medicine are also used in a therapeutic intervention, where livestock is treated when a disease is already present (details on these plants are not presented here ([22] for details)).

#### Care of the animals

Peppermint oil is used to clean the udder and teats of milking cows. When animals are given a wash, a yellow soft soap (*Schmierseife*) is used which is subsequently rinsed with water to which some apple cider vinegar is added.

#### Human-animal relationship

For the category human-animal relationship, one practice was mentioned that could be explained as deepening the friendship with an animal by using treats (bread).

#### Environmental management

Bunches of *Melissa officinalis* L. or *Juglans regia* L. in particular or together with *Mentha* spp. and/or *Sambucus nigra* L., for example, are put up in the stable to scent the air and hamper the development of insects, especially flies. Apple cider vinegar is not only given to animals as fodder but also vaporised in the stable. Various blessed herbs or *Juniperus communis* L. or *Salix* spp. are fumigated in the stable to prevent diseases in general. *Pteridium aquilinum* (L.) Kuhn is used as bedding material for chickens and moss is placed in pig stys to prevent swine erysipelas. Bedding with *J. regia* is said to be healthy in general and sawdust is said to have a negative effect, especially on young animals, because they always nibble on the bedding. Spruce (*P. abies*), larch (*L. decidua*), ash (*F. excelsior*), fir (*A. alba*) and straw are mentioned as good bedding material in addition to their uses as fodder.

#### Household remedy (excluding fodder medicine)

To prevent a retained placenta, different practices were mentioned: tincture of mountain arnica (*Arnica montana* L.) or hard liquor is used as an unction on the back of the cows; scalded hay-blossoms are put in a burlap bag and, while still hot, put on the back of cows; cows are rubbed with straw. *Hordeum vulgare* L., *L. usitatissimum*, *Matricaria chamomilla* L. or *Salix* spp. are used to administer a clyster after artificial insemination to maintain and improve pregnancy. To prevent swine erysipelas, swine wallow in the earth underneath *S. nigra*.

#### Cultural/religious activities

As a manifestation of religious practices based on Catholic beliefs, blessed herbs given on certain days were mentioned. Different medicinal plants, blessed on Mariä Himmelfahrt (Feast of the Assumption, 15 August), should prevent animals from contracting diseases in general and protect them from being hit by lightning and other such dangers. This mixture of plants is fed to the animals on the day on which the herbs are blessed, on the nights leading up to Christmas, on New Year’s day and at Epiphany, and also before animals go up to the alpine pastures. For the same reason, the catkins of blessed willow (*Salix* spp.) are fed to animals on Palm Sunday, with bread and salt or the peelings of the blessed horseradish (*Armoracia rusticana* P. Gaertn., B. Mey. & Scherb.).

### Details of knowledge of the kind of fodder/feed

Knowledge of plants that help maintain livestock health and welfare showed that, for the informants, fodder and feeding practices were key determinants of preventive measures (Table 4). As pointed out earlier, the relationships between fodder and livestock health and welfare are diverse, and details of this connection merit special mention.

#### Good/bad fodder and functional fodder

To maintain and/or increase the health and overall performance of the animals, it is essential that the animals demonstrate “appropriate” digestion according to those interviewed. Digestive processes are therefore evaluated by inspecting the dung. The consistency of a fresh cowpat has the look of a traditional flat loaf of bread: not too liquid, but not too dry and firm. To enhance and improve digestion in general, several plants were mentioned. The most frequently mentioned plants were linseed (*L. usitatissimum*), dried and ground needles of common spruce (*P. abies*), as well as dried or fresh entire stinging nettle plants (*Urtica dioica* L.) and the decoction of common juniper berries (*J. communis*).

Informants considered *U. dioica* not only to be digestive, but also to be of good quality in a general alimentary way and very nutritious, not just for cattle (whose considerable values were, according to the informants, a great improvement in the animal’s general appearance, especially obvious in a glossy coat, improved milk yields and an increase in milk fat), but for pigs and chickens too. Nettle, when chopped and blanched, is said to serve as fattening fodder for pigs. It was also mentioned as improving the performance of laying chickens and the condition of eggshells. The same use was mentioned for different taxa of dock (*Rumex* spp.). A practice to improve milk fat in the past was to feed the livestock nettle together with the chopped or stamped young sprouts and berries of common juniper (*J. communis*) and different taxa of thistles (e.g. *Cirsium spinosissimum* (L.) Scop., *Carlina acaulis* L.) and thyme (*Thymus* spp.). According to the informants, this was an excellent supplementary fodder in the summer, especially for dairy cows when they were up in the alpine meadows. Collecting and preparing this fodder was a large amount of work, particularly the destroying of thistle leaf prickles, which had to be beaten up or crushed in a mill. In winter, fresh chopped turnips (*Brassica rapa* var. *rapa* L.), which were stored in cellars, were fed to lactating dairy cows to improve milk yields. Today this traditional alpine crop plant is rarely cultivated and no longer used as fodder. Fodder beet (*Beta vulgaris* L.), which is purchased rather than cultivated in the region, is still used for this purpose.

A link between fodder and skin health, udder health and hoof quality of livestock was mentioned several times (in correlation with linseed, nettle and others, Table 4). Iceland moss was mentioned most. Traditionally Iceland moss (*C. islandica*) is not only used therapeutically, but is also seen as a nutritious and easily-digested fodder that improves not only the health of the animals’ skin, but also their health and fitness in general. In addition, it was mentioned that it contributes to weight gain and was used as a fattening fodder for pigs and bullocks, and as a restorer after diseases. Hay-blossoms were and are still used as concentrated feed and protein sources for cattle and fattening fodder for pigs. Stewed hay-blossoms mixed with grains are used to improve the laying performance of chickens. In addition, common chickweed (*St. media*), dead nettle (*Lamium* spp.), dock (*Rumex* spp.) and comfrey (*Symphytum officinale* L.) are mentioned as fodder with good feeding value for chicken and chicks.

Other plants mentioned as good quality fodder and healthy in general for ruminants (without specifying why) are dried leaves (leaf hay) of common ash (*F. excelsior*), oak (*Quercus* spp.), raspberry (*R. idaeus*), cherry (*Prunus avium* L.), common hazel (*C. avellana*) as well as the needles of fir (*A. alba*) and larch (*L. decidua*). Good fodder quality for pigs includes potato (*Solanum tuberosum* L), fodder beet (*B. vulgaris*), turnip (*B. rapa* var. *rapa*) and pumpkin (*Cucurbita* spp.). *Usnea* spp. and *Elymus repens* (L.) Gould were used when fodder was scarce. To improve the physical shape of animals in general, the juice of birch (*Betula* spp.) was mentioned.

Plants such as *Rhinanthus* spp. and *Ranunculus* spp. were reported to have negative effects if represented in a fairly large quantity in meadows or grazing grounds. Green alder (*Alnus alnobetula* (Ehrh.) K.Koch and alpine rose (*Rhododendron* spp.) were mentioned as weeds in alpine grassland. Three plants were reported as having a negative impact, but were also welcomed when cut and carried to the animals as “fresh” fodder (*Anthriscus sylvestris* (L.) Hoffm.) or as fodder prepared specifically for a particular animal species (*Rumex* spp. for chicken) or used in small quantities (*Achillea millefolium* L.).

In general, informants addressed the effects of the form of presentation (fresh, dry, cooked, chopped etc.) or the methods of improving food substance on increasing the fodder’s acceptability by livestock. This was mentioned in particular for Iceland moss, which has to be cooked, hay-blossoms, which have to be brewed, or spruce needles, which have to be dried and ground before being presented to livestock. In addition, the mode of harvesting is essential for good quality and acceptability. In former times, hay-blossoms were never contaminated with soil because all of the working steps were performed manually, from harvesting to cleaning, with a special coarse-meshed sieve. However, hay exclusively produced by hand is an exception nowadays.

Informants pointed out that it is important to note that the acceptability of a particular type of fodder (e.g. dried stinging nettle, meal of spruce needles) is due to the fact that animals have become used to it, and that the ration of this functional fodder has to be carefully composed otherwise it could be toxic (e.g. animals must not be fed too many ground spruce needles).

#### Fodder medicine

Reduced appetite, which is digestive in origin and does not correlate with a serious disorder or pain (e.g. teeth problems), is treated with several plants. The most important one for stimulating digestion is wormwood (*A. absinthium*), which is used mainly for cows. The whole plant can be eaten either fresh or dried. The chopped or stamped sprouts and berries of common juniper (*J. communis*) or fresh thyme mixed with concentrated feed are used in a similar fusion. Decoctions containing common yarrow (*A. millefolium*), chamomile (*M. chamomilla*), marigold (*Calendula officinalis* L.), wormwood, juniper and dwarf mallow (*Malva neglecta* Wallr.) were said to be good for digestion and also had a calming effect. These plants can be used either alone or together.

To regulate digestion when the dung is too fluid, feeding the animal alpine meadow hay (*Bergheu*, acquired at a height of 1900 m above sea level and higher) was mentioned most. Other solutions are wheat bran and leaf hay. Alpine meadow hay is, in addition, considered to be very healthy for overall vitality and fitness, and as palatable and easily digested by livestock, but it is not useful for enhancing livestock performance. It is used only in small amounts and seen as “medicinal”, and not as fodder like hay, which is harvested near the homesteads. This is also due to the low quantity of alpine hay that can be harvested. In this context, informants mentioned a correlation between the composition of fodder, efficient digestion and reproductive performance. Feeding livestock alpine meadow hay is said to prevent disorders in fertility in general. The same is said for leaf hay.

Regarding fertility, liquid from fermented cabbage (*Sauerkrautsaft, Brassica oleracea* var*. capitata* L.) or apple cider vinegar is given together with concentrated feedingstuffs in order to avoid silent oestrus or as an oestrus inducer. Sprouting grains of rye (*Secale cereale* L.) mixed with yeast are also used. In this context, it was emphasised that sprouted grains are more useful than the dried ones. Grains of *Avena sativa* L. either boiled or macerated are fed to animals. Buckwheat (*Fagopyrum esculentum* Moench) is fed to livestock to bring mares in season (to facilitate ovulation). Tea of lady’s mantle (*Alchemilla* spp.) and cranesbill (*Geranium* spp.) was mentioned as being good for female organs and is given before the females are inseminated (bred) to ensure better acceptance of a foetus.

Many practices relate to pregnancy. Special attention is given to the nutrition of the pregnant animal. Linseed (*L. usitatissimum*) or a combination of linseed and different ingredients, such as wheat bran, barley, oat or rye, eggs, chamomile and/or Iceland moss, is fed to the animals during the last three weeks of pregnancy to avoid problems in general.

As preparatory fodder to facilitate delivery, liquid from fermented cabbage and linseed are mentioned. Seeds of *Cannabis sativa* L. are mixed with butter as a labour inducer, and to stimulate uterine contractions. Raw onion (*Allium cepa* L.), sometimes in combination with lard, is administered as fodder to widen the birth canal and help with the quick expulsion of the placenta. Also, as a preventive cleansing agent, a decoction of common yarrow (*A. millefolium*), caraway (*Carum carvi* L.), fennel (*Foeniculum vulgare* Mill.) and the oily extraction of St. John’s wort (*Hypericum perforatum* L.) are used. Another mixture is boiled Iceland moss, linseed and barley. For the same purpose, tincture of mountain arnica is given internally in small amounts.

After birth, a strong coffee or black tea is sometimes mixed with schnapps or eggs and butter and given to cows and/or calves as refreshment and to avoid circulatory disorders. Nettle seeds (*U. dioica*) are mentioned as a restorer not only after delivery, but also after any disease, especially for horses. A decoction of hay-blossoms is said to be helpful in general.

To avoid milk fever, besides the feed already mentioned for pregnancy, a boiled mash of dock (*Rumex* spp.), Iceland moss and skimmed milk or a broth of hay-blossoms is fed to the animals at this time.

For the prevention of bovine influenza and to improve health and fitness of cows in general, the berries from junipers and Iceland moss are combined with black tea and schnapps or a concentrate of elderberry berries (*S. nigra*) and fed to cattle. Apple cider vinegar or vinegar produced from fermented turnips (*B. rapa* var. *rapa*, *Rübenkrautessig*) is given during the winter housing period in the watering trough or with concentrated feed once a week. An infusion of thyme is sprayed in the mouth and on the nose of cattle. Also, for prevention, the oily extraction of St. John’s wort (*H. perforatum*) is mixed with concentrated feeding stuff or mixed into milk for calves and fed to cows. To avoid swine erysipelas, a slurry of stinging nettle and hay-blossoms is used. Newly hatched chicks are fed a decoction of chamomile (*M. chamomilla*) and burnet saxifrage (*Pimpinella saxifraga* L.) to give them a good start and prevent diseases. Avoiding stress is said to minimise the outbreak of illness, including bovine influenza. Here, in addition to management practices, some plants that can be used in this context were also mentioned. When calves are bought in or transported, chamomile or mint tea is administered as a sedative and helps prevent bovine influenza. To avoid irritating the animals and as a type of sedative in general, oat cereal (*A. sativa*), wormwood leaves (*A. absinthium*), sunflower seeds *(Helianthus annuus* L.) and hard liquor were listed.

Good nutritional support and reduction of stress may, according to the informants, also contribute to preventing intestinal parasites. Feeding animals branches of common spruce (*P. abies*) for a period of at least one or two weeks before ruminants are driven up to the alpine pastures and in autumn before they are brought into the winter housing, is said to be an adequate diet to protect the animals against parasites. Feeding livestock dry or fresh bracken (*P. aquilinum*) in very small amounts was also mentioned in this context. When deworming with anthelminthics, a supplementary feeding of stinging nettle contributes to normalising and calming the digestive tract.

## Discussion

The success of domestic livestock farming depends on animal health and welfare. Farmers’ attitudes and the attention given to their herds appear to be crucial success factors in herd health and welfare situations. These success factors are key determinants of the animals’ reproductive and growth rates [8, 43]. The informants in this study viewed fodder and feeding as the most important preventive measures for ensuring animal health and welfare. That feeding is central to the welfare of livestock is in line with the standard scientific literature on animal health and in organic farming as well [44–46].

Nutrition, among other factors, is an important element of ethnoveterinary methods in disease prevention and general health maintenance worldwide [13, 14, 21, 47–50]. By regularly consuming medicinal foods or food medicines, particularly wild greens, animals – like humans – also ingest important non-nutrient substances [50]. In fact, the value of ‘food as medicine’ for humans in the context of local knowledge has been widely assessed through research [42], but the potential health benefits of fodder and nutraceuticals [17] in ethnoveterinary methods require further attention [17, 42, 50]. Viewing a practice as nutritional or medicinal is often only a matter of definition. The boundary between food and medicine is rooted in emic cultural interpretations and is thus difficult to assess. The role of gathered food plants merits greater attention due to their biomedical value and their socio-economic importance. A veterinary governmental regulation was implemented that aims to legally distinguish between plants that are either to be fed or applied as medicine [51, 52]. This regulatory differentiation is seen by the authors as an artefact.

However not every traditional practice can be said to be based on pharmacological evidence or effective in every condition, and results of ethnoveterinary research have to be critically discussed bearing in mind the recent results of pharmacological, toxicological and clinical studies before they can be recommended for wider practice. A considerable proportion of the documented uses of plant taxa is in accordance with established pharmacological effects [27].

Informants generally assumed that feeding strategies in the past were healthier because of the previous diversity of high-quality local feeding stuff from diverse feed sources and the mode of presentation. Overall, the plants most frequently mentioned in this study were traditional plants. Nonetheless it is obvious that although people in the region still have knowledge about the use of certain traditional plants, their actual use is continuing to decrease or has already disappeared altogether. Regular feeding with tree fodder is widely seen as being beneficial for animals [53], especially in the context of tannin-rich forages and intestinal parasites [54–56]. In Eastern Tyrolean feeding practices, the use of dried and grounded spruce needles (*P. abies*) as concentrated feeding stuff for example was common and highly recommended for animal health and welfare, but this feeding practice was only practised by one farmer at the time of the survey.

Feeding spruce needles might reduce emissions of methane as they are known to enhance and improve digestion. There is considerable discussion about the correlation between feeding practice and methane emissions [57, 58]. The value of such feeding techniques should be assessed in greater detail and might contribute to climate change mitigation. Spruce could also be considered as one of the plants with economical potential for the study area. Spruce needles and branches are often seen as a waste product and are accumulated in great quantities in the forest. This raw material would be locally available in larger quantities and inexpensive, however it's harvesting and preparation would present a challenge if this fodder resource were to be incorporated into “modern” animal farming at all. Plieninger and Wilbrand argue that there is evidence to suggest that labour-intensive techniques, such as manual handling for providing traditional fodder resources, will be a crucial factor in their future viability [59].

Generally the potential of unconventional plants is increasingly being identified in the context of underutilised crops for human food [60, 61] but not for animal fodder, and this would be of huge importance to the organic farming movement [62]. For example nettles, one of the plants mentioned most frequently in this study, are among the most undervalued economic plants [60, 63, 64] and attract little attention in scientific research as a fodder plant with just a few exceptions [21, 65].

Global research on underutilised crops shows that the use of traditional fodder plants is declining because of two factors: they cannot easily be harvested mechanically and wheat (or other grain or soya) is easier to use in their place [61]. In the present study, grain feeding (barley and oats) was mentioned by the informants as favouring animal health and welfare, being recommended prior to breeding and being required only in low quantities. This traditional feeding practice is different from the massive use of grain feeding, material fit for human consumption, which plays such an important role in industrialised agriculture [3, 66]. Massive grain feeding is discussed as having a negative impact on animal welfare, with increasing economic relevance also due to the resulting disorders [3, 67] and organic farming research projects such as “feed not food” [68]. Unlike other farming systems, external inputs are clearly limited in organic farming and adaptation to local conditions is needed, where feed sources must be seen as part of the agroecologial system [44, 69] and not as the separation of agriculture from the local environment [70]. Hence knowledge of traditional feeding practices could be of great importance. Moreover while recent animal nutrition science has focused on the impact of feed on animal growth and performance and on product quality, the health aspect becomes even more important in organic farming [71].

Besides favouring some currently neglected and underutilised fodder crops, local conditions also have to be assessed, such as the abundance and occurrence of plants or their conservation status. If wild plants are rarely available, for example, the possibility of cultivation might be examined. It is not sensible to recommend gathering wild fodder plants to promote animal health and welfare while causing harm to the plant population and the wider environment.

Treatments of fodder preparations and their effects on nutritive and dietary value, efficiency and safety etc. have been evaluated in several scientific studies for common fodder [72] and have to be considered in the evaluation of traditional, local fodder resources e.g. including fodder from trees [53]. They have to be given particular consideration because past knowledge is being transformed and lost in the rapid process of acculturation faced by traditional societies [13, 17].

## Conclusions

This study shows that farmers’ local and traditional ethnoveterinary knowledge of animal health and welfare is of importance to the organic farming movement. Although there is no desire by the authors to generalise, this knowledge deserves more attention. Future studies have to prove or disprove the sufficiency of individual practices, nevertheless local knowledge is clearly one of the starting points for the further development of sustainable animal health care programmes.
